# ASSISTED LIVING STAFF VIEWS OF FAMILY INVOLVEMENT IN PALLIATIVE CARE FOR RESIDENTS WITH DEMENTIA

**DOI:** 10.1093/geroni/igae098.2994

**Published:** 2024-12-31

**Authors:** Jessica Yauk, Debra Dobbs, Lindsay Peterson

**Affiliations:** University of South Florida, Tampa, Florida, United States; University of South Florida, Tampa, Florida, United States; University of South Florida, Tampa, Florida, United States

## Abstract

Of the close to 300,000 residents in assisted living (AL) living with dementia, an estimated 40% will die there and be in need of end-of-life care. Family of residents living with dementia are an important part of the care convoy in AL. This qualitative study aimed to understand staff perspectives about family involvement in end-of-life care. The Palliative Care Education in Assisted Living for Dementia Care Providers intervention study (2019-21) included a sample of 17 AL staff from six treatment sites in Florida who completed four homework assignments. Two of the assignments were writing assignments that 1) asked about a good death for residents and 2) asked staff specifically to identify one resident with dementia who they could do one thing to make them comfortable. A priori codes were used based on the physical, emotional, social and spiritual care domains of palliative care asked about in the homework assignments. The data was coded using Atlas.ti version 23. Family was involved in all four domains of palliative care. Social and emotional aspects of care were the most prominent (e.g., playing music, visits, care coordination, delivery of food) followed by spiritual care (e.g., pray together, attend religious services). Physical care tasks by family were reported less often. Less physically demanding care tasks were performed by the family (e.g., grooming, dressing) with family reliance on direct care staff and hospice providers for more physically demanding care (bathing, transfers), and for pain management. We will discuss the role family play in AL care provision.

